# Supplementary material for: The Candida albicans transcription factor Cas5 couples stress responses, drug resistance and cell cycle regulation
Source: Nat Commun. 2017 Sep 11;8:499. doi: 10.1038/s41467-017-00547-y (PMC5593949; doi:10.1038/s41467-017-00547-y)
Supplement: Supplementary file 1 — Supplementary Information [file 41467_2017_547_MOESM1_ESM.pdf]

Description of Supplementary Files

File Name: Supplementary Information
Description: Supplementary Figures, Supplementary Tables and Supplementary References

File Name: Supplementary Data 1
Description: RNA PolII ChIP-seq data comparing wild type and <i>cas5Δ/cas5Δ</i> mutant strains under basal condition.

File Name: Supplementary Data 2
Description: RNA PolII ChIP-seq data for the wild type strain comparing basal and caspofungin treatment conditions.

File Name: Supplementary Data 3
Description: RNA PolII ChIP-seq data for the <i>cas5Δ/cas5Δ</i> mutant strain under basal and caspofungin treatment condition.

File Name: Supplementary Data 4
Description: RNA PolII ChIP-seq data comparing wild type and <i>cas5Δ/cas5Δ</i> mutant strains under caspofungin treatment condition.

File Name: Supplementary Data 5
Description: Common and unique differentially bound genes between the comparisons of the <i>cas5Δ/cas5Δ</i> mutant to the wild type strain and of wild type strain in the presence and absence of caspofungin treatment.

File Name: Supplementary Data 6
Description: Cell cycle expression pattern of genes differentially bound in <i>cas5Δ/cas5Δ</i> mutant in comparison to the wild type strain under basal caspofungin treatment conditions.

File Name: Supplementary Data 7
Description: Physical interactors of Cas5 identified by co-immunoprecipitation coupled with mass spectrometry.

File Name: Peer Review File

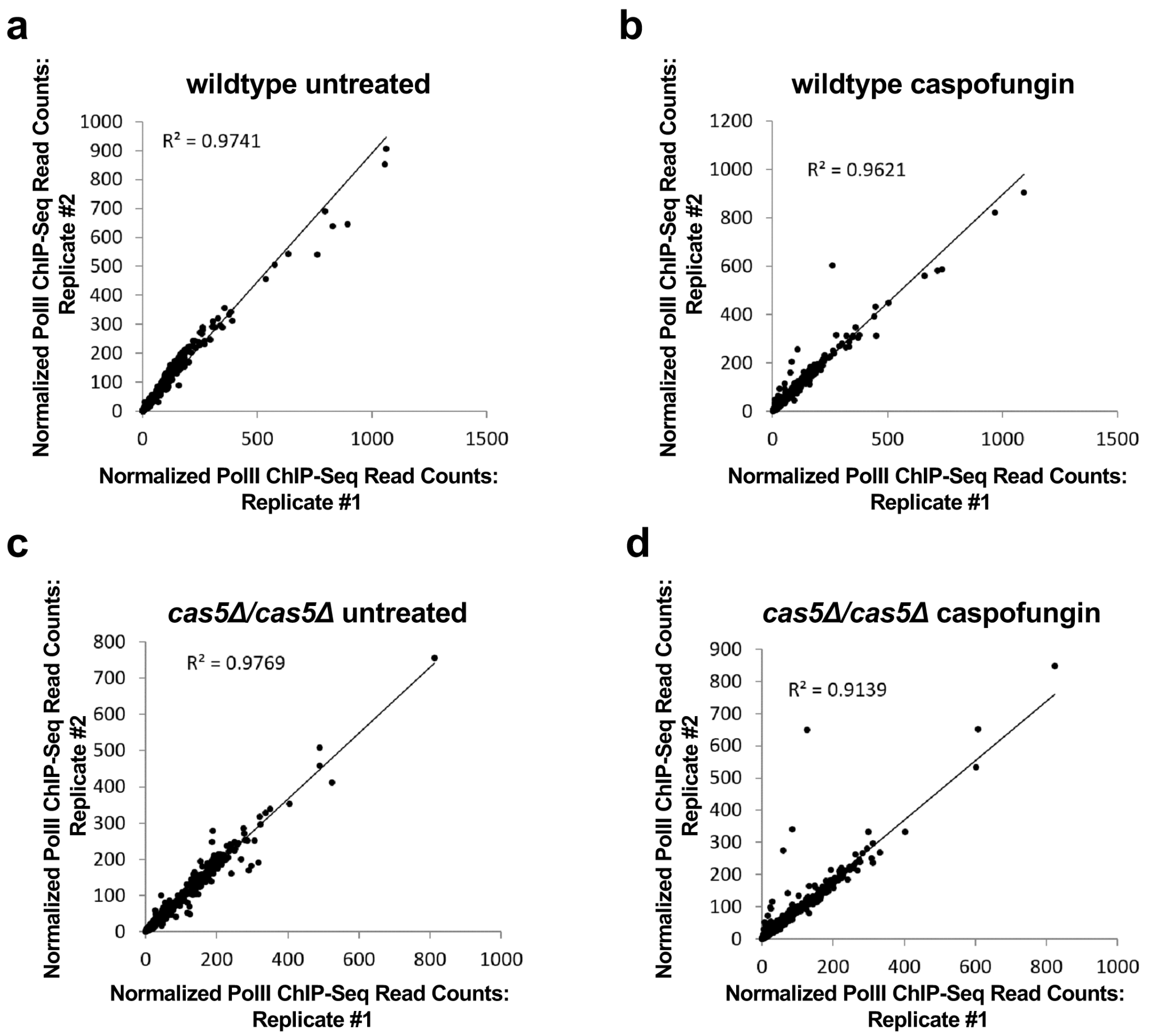

Supplementary Fig. 1: Correlation plots for RNA PolII ChIP-Seq analysis. Scatterplots highlighting the correlation of normalised PolII ChIP-Seq read counts between biological replicates of PolII ChIP-Seq experiments for **(a)** wild type untreated, **(b)** wild type with caspofungin, **(c)** *cas5*Δ/*cas5*Δ mutant untreated, and **(d)** *cas5*Δ/*cas5*Δ mutant with caspofungin.

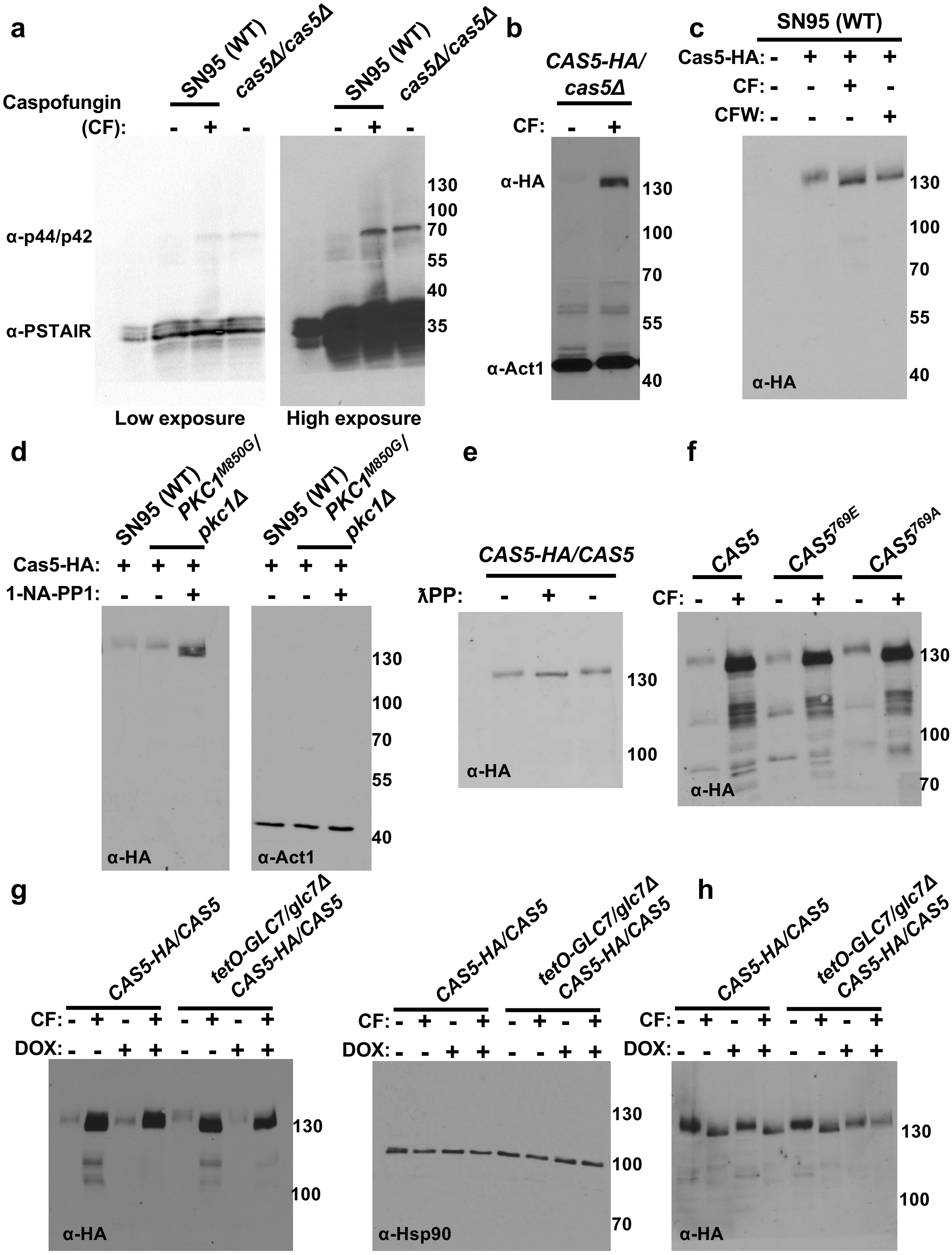

a
SN95 (WT)
cas5Δ/cas5Δ
Caspofungin (CF):
α-p44/p42
α-PSTAIR
Low exposure
High exposure
130
100
70
55
40
35
b
CAS5-HA/cas5Δ
CF:
α-HA
α-Act1
c
SN95 (WT)
Cas5-HA:
CF:
CFW:
α-HA
d
PKC1M850G/pkc1Δ
Cas5-HA:
1-NA-PP1:
α-HA
α-Act1
e
CAS5-HA/CAS5
λPP:
α-HA
f
CAS5
CAS5769E
CAS5769A
CF:
α-HA
g
CAS5-HA/CAS5
tetO-GLC7/glc7Δ CAS5-HA/CAS5
CF:
DOX:
α-HA
α-Hsp90
h
α-HA

Supplementary Fig. 2: Images of complete Western blots shown in main figures. (a) Deletion of *CAS5* leads to the activation of the cell wall integrity pathway in the absence of cell wall stress. A SN95 wild-type strain and a *cas5Δ/cas5Δ* mutant were left untreated (-) or treated for 1 hour with 125 ng/ml of caspofungin (+), as indicated. Phosphorylated Mkc1 was monitored by Western blot and detected with α-p44/42 antibody. **(b)** Cas5 increases in response to caspofungin. Levels of Cas5 were monitored by Western blot and detected with an α-HA antibody. Actin was detected with an α-β-actin antibody as a loading control. **(c)** Cas5 is post-translationally modified upon cell wall stress treatment. Cells were grown to log phase and subsequently treated with 125 ng/ml of caspofungin or 50 μg/ml of calcofluor white for 1 hour. Total protein was resolved by SDS-PAGE and the blot was hybridized with an α-HA to monitor Cas5 migration. **(d)** Cas5 is post-translationally modified upon Pkc1 inhibition. Cas5 migration and actin detection were monitored as part **b**. **(e)** Cas5 is phosphorylated in the absence of stress. Cas5 migration was monitored by Western blot and detected with an α-HA antibody. Treatment of protein lysate with lambda phosphatase resulted in a faster migrating band, indicative of a loss of phosphate groups. **(f)** Phosphomutations in *CAS5* do not affect band shifts associated with activation of the cell wall stress response, as observed upon caspofungin treatment. Cas5 was monitored by Western blot and detected using an α-HA antibody. **(g)** Upregulation of Cas5 expression does not depend on Glc7. *CAS5-HA/CAS5* and *CAS5-HA/CAS5 tetO-GLC7/glc7Δ* strains were cultured in the absence or presence of doxycycline and caspofungin, as indicated. Cas5 was monitored by Western blot and detected with an α-HA antibody. Hsp90 protein levels served as a loading control. **(h)** Post-translational modification of Cas5 is absent upon *GLC7* depletion. The Western blot was performed as described in **b,** except caspofungin treated samples were diluted 5-fold to achieve equal loading of Cas5

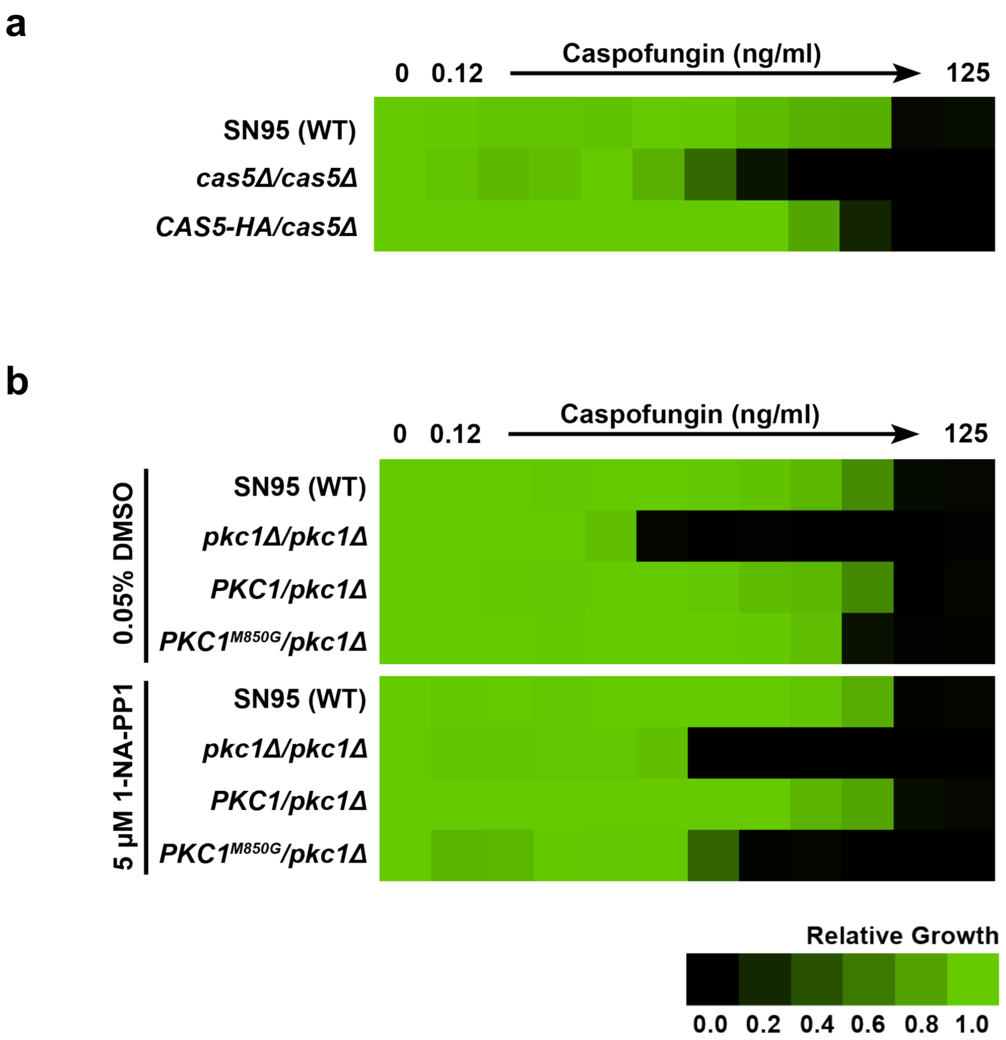

Supplementary Fig. 3: Genetic validation of mutant strains. (a) HA-tagged allele of *CAS5* is functional. Caspofungin susceptibility assays were conducted in YPD medium. Growth was measured by absorbance at 600 nm after 48 hours at 30°C. Optical densities were averaged for duplicate measurements. Data was quantitatively displayed with colour using Treeview (see colour bar). **(b)** ATP analogue 1-NA-PP1 specifically inhibits Pkc1 kinase activity. Caspofungin susceptibility assays were conducted in YPD medium in the presence of 0.05% DMSO or 5 µM

1-NA-PP1. Growth was measured by absorbance at 600 nm after 48 hours at 30°C. Data was analyzed and plotted as in part **a.**

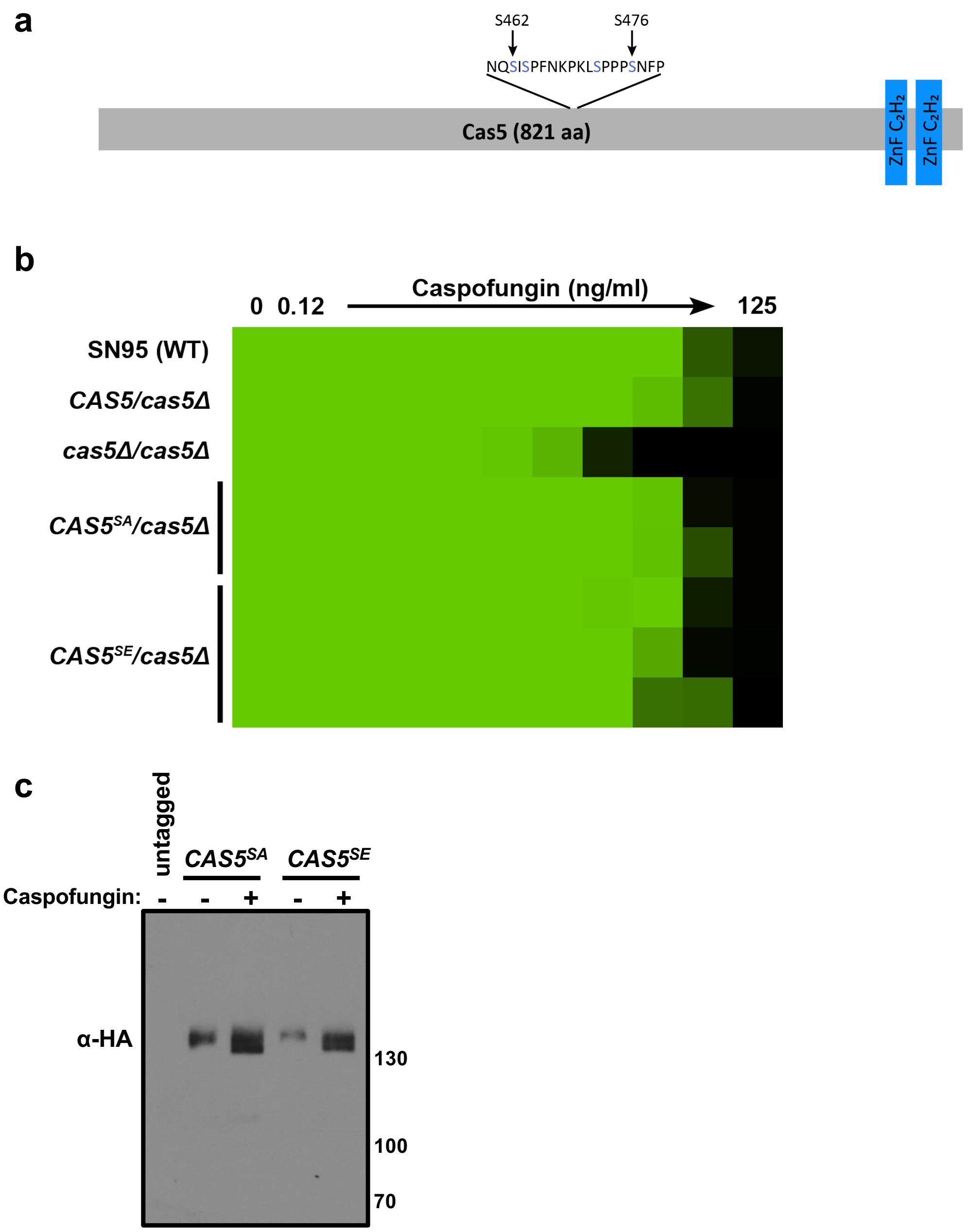

Supplementary Fig. 4: The phosphorylation sites identified by mass spectrometry are not sufficient for regulation of Cas5 function in response to cell wall stress. (a) A schematic showing the phosphorylated serine residues identified in Cas5 by the mass spectrometry analysis. **(b)** Both phosphomimetic and phosphodeficient substitutions of the Cas5 serine residues did not affect caspofungin tolerance. The phosphomimetic (SE) and phosphodeficient (SA) alleles of *CAS5*, consisting of four substitutions at serine residues 462, 464, 472, and 476, were introduced into a *cas5*Δ/*cas5*Δ mutant individually as the only *CAS5* allele in the strain. Caspofungin susceptibility assays were conducted in YPD medium. Growth was measured by absorbance at 600 nm after 48 hours at 30°C. Optical densities were averaged for duplicate measurements. Data was quantitatively displayed with colour using Treeview (see colour bar in Fig. S2). **(c)** The phosphomimetic (SE) and phosphodeficient (SA) substitutions of the serine residues on Cas5 do not affect the band shift associated with activation of the cell wall stress response. Cells were subcultured in YPD for 3 hours to reach log phase, and subsequently treated with 125 ng/ml of caspofungin for 2 hours. Levels of Cas5 were monitored by Western blot and detected with an α-HA antibody. Full blot is shown in the Figure.

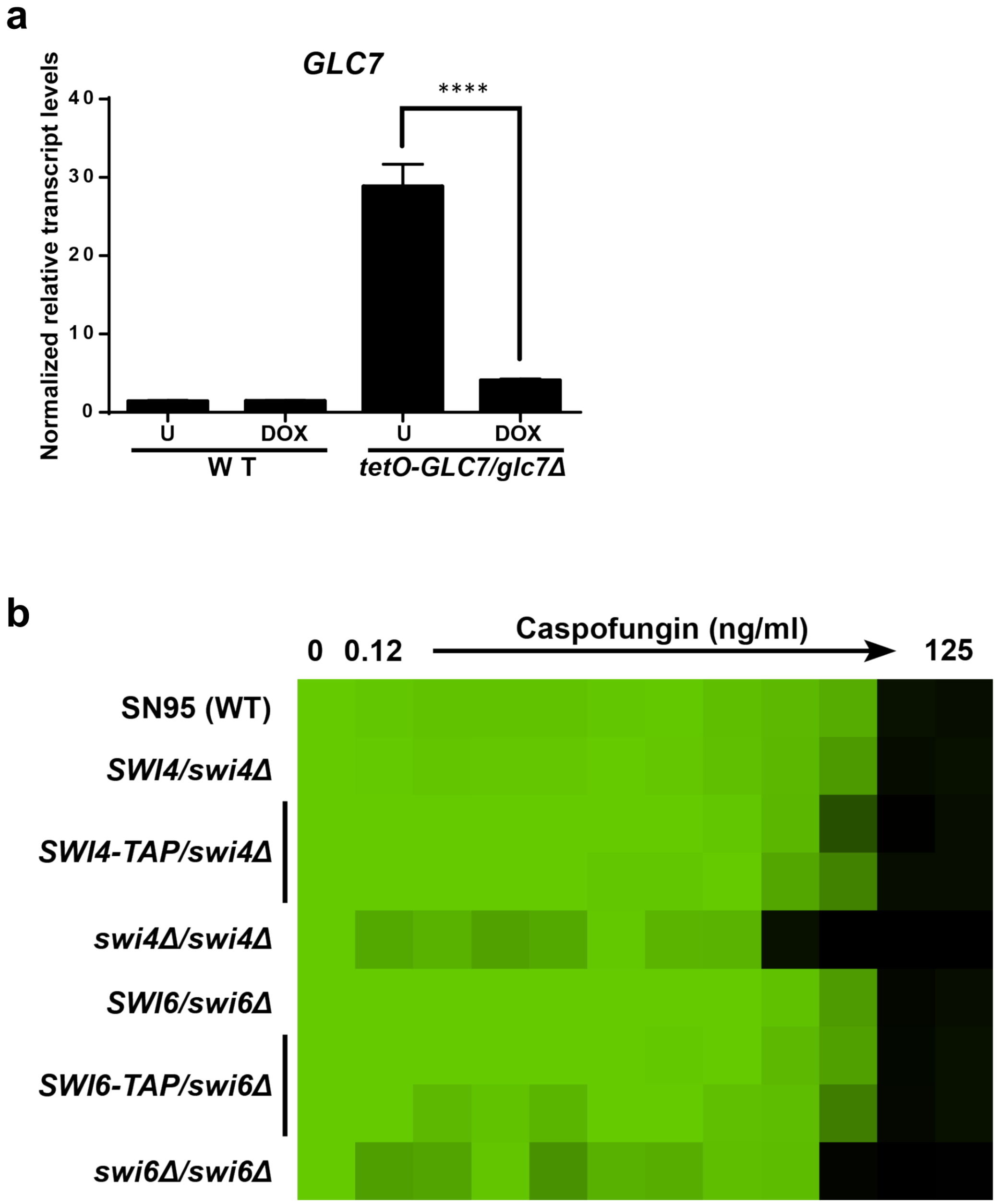

Supplementary Fig. 5: *GLC7* is depleted upon doxycycline treatment in the *tetO-GLC7/glc7*Δ strain, and the TAP tagged versions of Swi4 and Swi6 are functional. (a) Wild-type and *tetO-GLC7/glc7*Δ strains were grown for 24 hours in the presence or absence of 0.02 µg/ml of doxycycline. The strains were subcultured again in the same conditions for 3 hours and subsequently treated with or without caspofungin for 1 hour. The transcript level of *GLC7* was

monitored by qRT-PCR and normalized to *GPD1*. Error bars represent standard deviation (s.d.) from the mean of triplicate samples. Levels of *GLC7* upon transcriptional repression with doxycycline were compared using Tukey's multiple comparisons test in GraphPad Prism (**** $P<0.0001$). **(b)** Caspofungin susceptibility assays were conducted in YPD medium. Growth was measured by absorbance at 600 nm after 48 hours at 30°C. Optical densities were averaged for duplicate measurements. Data was quantitatively displayed with colour using Treeview (see colour bar in Fig. S2).

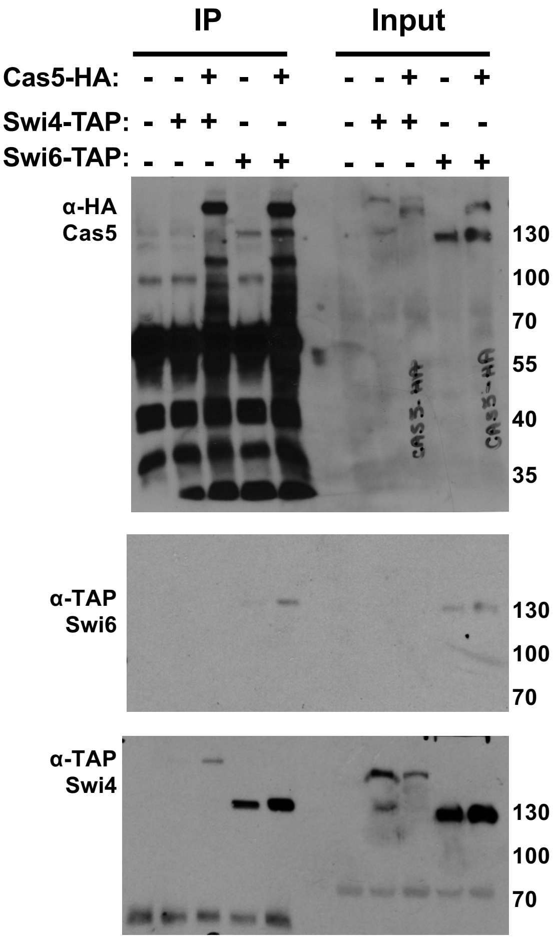

.

Supplementary Fig. 6: Images of complete immunoprecipitation Western blots shown in Figure 8. Swi4 and Swi6 co-purify with Cas5. C-terminally HA-tagged Cas5 was immunoprecipitated with α-HA beads. Swi4 and Swi6 co-purification was monitored by Western blot and detected with an α-TAP antibody. Cas5 pull down was confirmed with detection using an α-HA antibody. Input samples confirm the expression of tagged proteins. Top panel, middle panel, and bottom panel represent full blots used for corresponding panels in Figure 8.

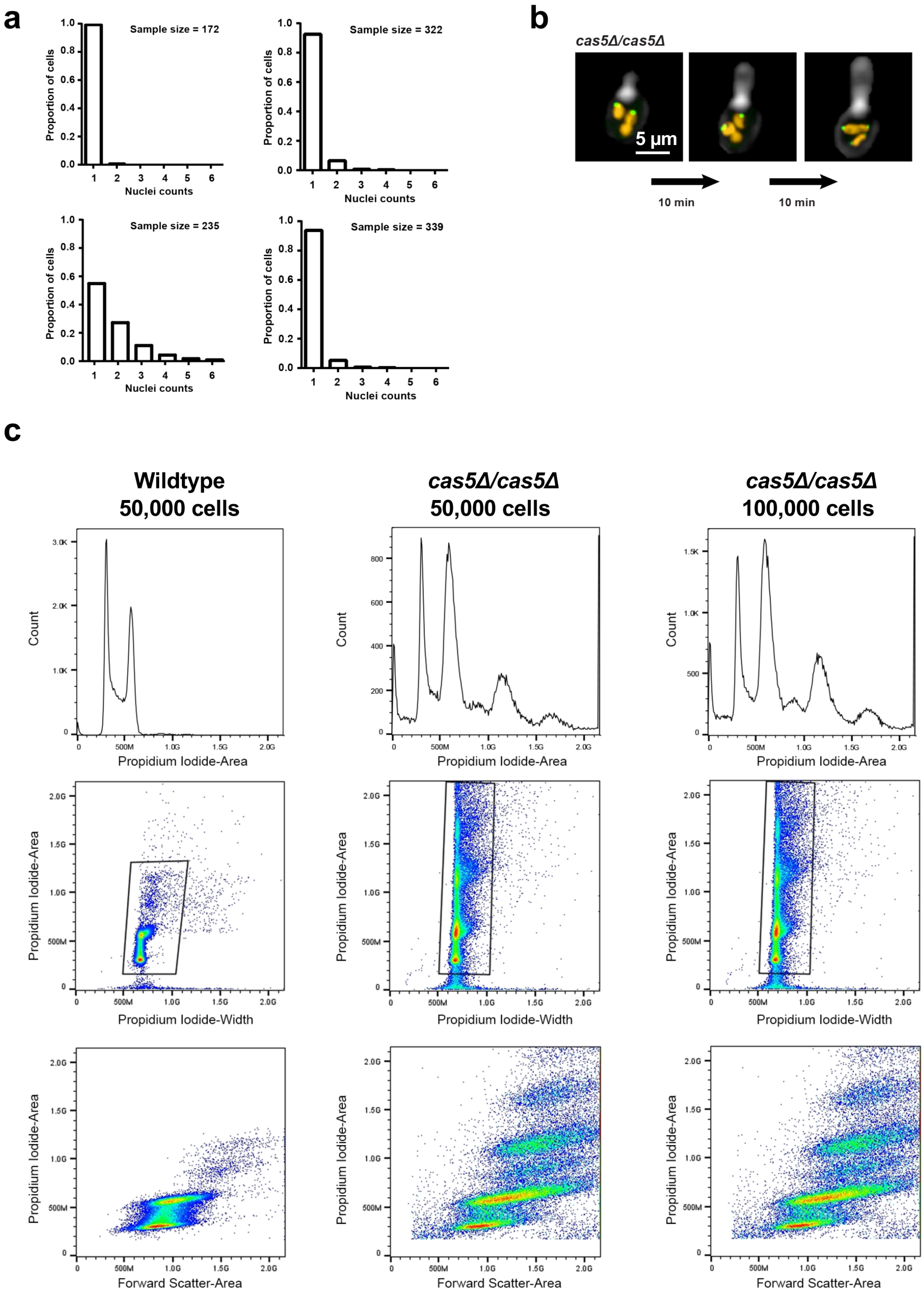

a
Sample size = 172
Sample size = 322
Sample size = 235
Sample size = 339
Proportion of cells
Nuclei counts
b
cas5Δ/cas5Δ
5 μm
10 min
10 min
c
Wildtype
50,000 cells
cas5Δ/cas5Δ
50,000 cells
cas5Δ/cas5Δ
100,000 cells
Count
Propidium Iodide-Area
Propidium Iodide-Width
Forward Scatter-Area

Supplementary Fig. 7: Cas5 regulates proper nuclear division. (a) Histograms highlighting the number of nuclei present per cell in wild-type, *cas5Δ/cas5Δ, swi4Δ/swi4Δ,* and *swi6Δ/swi6Δ* strains. Plotted are results from a representative biological replicate. A minimum of 172 cells were counted for each strain. **(b)** Mitotic spindles are misaligned during cell division in a mutant lacking Cas5, monitored as in Fig. 9. Scale bar represents 5 μm. **(c)** Cellular DNA content measured by propidium iodide and flow cytometry of the (left panel) wild-type diploid (SN95), and the (middle and right panels) *cas5Δ/cas5Δ* mutant strain. Either 50,000 cells (left and middle panels) or 100,000 cells (right panel) were analyzed per strain. No difference was detected between the 50,000 or 100,000 cell populations. The top row of each column is the number of cells (Count) plotted by propidium iodide flurorescence (Propidium Iodide-Area). This is to rule out the possibility that the increased ploidy levels in the *cas5Δ/cas5Δ* mutant were due to cell aggregates. In the middle row, aggregates were detected by Propidium Iodide-Area versus Propidium Iodide-Width. Because single cells (G0/1 or G2/M) will have similar pulse width values (or transit time across the laser beam), we observed a near vertical line on the Propidium Iodide-Width axis that represents the width of a singlet population. Aggregates, however, will have larger width values and are detected only minimally to the right of the singlet population (> 1.0G). Furthermore, in the *cas5Δ/cas5Δ* mutant strain, the fluorescence intensity of the Propidium Iodide-Area increased with the ploidy intervals detected in the top row (indicated by red/orange/yellow). Using this singlet population gate, we then plotted the Propidium Iodide-Area by Forward Scatter-Area (bottom row). This plot shows that ploidy increases with cell size (Forward Scatter-Area), and this cell size increase is not due to cell aggregates because this population is derived from the singlet population.

Supplementary Table 1. ***Candida albicans*** **strains used in this study.**

Strain Name	Genotype	Source
CaLC191 (DAY185)	*pARG4::URA3::arg4::hisG/arg4::hisG pHIS1::his1::his1/his1::hisG*	1
CaLC1349	DAY185 *cas5::ARG4/cas5::URA3*	2
CaLC1350	DAY185 *cas5::ARG4/cas5 ::URA3 pCAS5::HIS1::his1::hisG/his1::hisG*	2
CaLC239 (SN95)	*arg4Δ/arg4Δ his1Δ/his1Δ URA3/ura3Δ::imm434 IRO1/iro1::imm434*	3
CaLC2034	SN95 *cas5::FRT/CAS5*	This study
CaLC2056	SN95 *cas5::FRT/cas5::FRT*	This study
CaLC2087	SN95 $FKS1^{F641S}$/*FKS1*	4
CaLC3908	SN95 $FKS1^{F641S}$/*FKS1 cas5::FRT/cas5::FRT*	This study
CaLC3909	SN95 $FKS1^{F641S}$/*FKS1 cas5::FRT/cas5::FRT*	This study
CaLC1255	*SN95 CaTAR-FRT pkc1::FRT/pkc1::FRT*	5
CaLC1256	*SN95 CaTAR-FRT pPKC1-FRT/pkc1::FRT*	5
CaLC3067	*SN95 CaTAR-FRT* $PKC1^{M850G}$*-FRT/pkc1::FRT*	6
CaLC3378	*SN95 CaTAR-FRT* $PKC1^{M850G}$*-FRT/pkc1::FRT CAS5-HA-HIS1/CAS5*	This study
CaLC3859	SN95 *CaTAR-FRT-tetO-CAS5/cas5::FRT*	This study
CaLC3113	SN95 *CAS5-HA-HIS1/cas5::FRT*	This study
CaLC3151	SN95 *CAS5-HA-HIS1/cas5::FRT*	This study
CaLC2213	SN95 *CAS5-HA-HIS1/CAS5*	This study
CaLC3044	SN95 *CAS5-HA-HIS1/CAS5-HA-ARG*	This study
CaLC4285	DAY286 *CAS5-HA-HIS/CAS5*	This study

CaLC3209	SN95 *CAS5*^{S769E}*-HA-HIS1/cas5::FRT*	This study
CaLC3189	SN95 *CAS5*^{S769A}*-HA-HIS1/cas5::FRT*	This study
CaLC4036	SN95 *swi4::FRT/swi4::FRT*	This study
CaLC4330	SN95 *swi6::FRT/swi6::FRT*	This study
CaLC3391	SN95 *SWI4-TAP-ARG4/SWI4*	This study
CaLC3393	SN95 *SWI6-TAP-ARG4/SWI4*	This study
CaLC3395	CaLC3151 *SWI4-TAP-ARG4/SWI4*	This study
CaLC3398	CaLC3151 *SWI6-TAP-ARG4/SWI6*	This study
CaLC3932	SN95 *CaTAR-FRT-tetO-GLC7/glc7::FRT*	This study
CaLC3952	CaLC3932 *CAS5-HA-HIS1/CAS5*	This study
CaLC3672	CaLC2056 *CAS5S462A/S464A/S472A/S476A-HA-HIS1/cas5::FRT*	This study
CaLC3673	CaLC2056 *CAS5S462A/S464A/S472A/S476A-HA-HIS1/cas5::FRT*	This study
CaLC3693	CaLC2056 *CAS5S462E/S464E/S472E/S476E-HA-HIS1/cas5::FRT*	This study
CaLC3694	CaLC2056 *CAS5S462E/S464E/S472E/S476E-HA-HIS1/cas5::FRT*	This study
CaLC3695	CaLC2056 *CAS5S462E/S464E/S472E/S476E-HA-HIS1/cas5::FRT*	This study
CaLC4471	SN95 *SWI4-TAP-ARG4/swi4::FRT*	This study
CaLC4499	SN95 *SWI6-TAP-ARG4/swi6::FRT*	This study
CaLC4705	SN95 *HHF1-RFP-NAT/HHFR DAD2-GFP-HIS/DAD2*	This study
CaLC4707	SN95 *cas5::FRT /cas5::FRT HHF1-RFP-NAT/HHFR DAD2-GFP-HIS/DAD2*	This study

Supplementary Table 2. Bacterial plasmids used in this study.

Strain Name	Description	Source
pLC49	*FLP-CaNAT, NATr, ampR*	[7]
pLC383	*GFP-HIS*, *ampR*	[8]
pLC435	*Clp1-ADH1-CHERRY, ampR*	[9]
pLC447	*CaCherry-NAT, NATr, ampR*	This study
pLC575	*pFA-HA-HIS1, ampR*	[10]
pLC576	*pFA-HA-ARG4, ampR*	[10]
pLC790	pLC49 *CAS5*$^{DBD\ domain}$*-HA-HIS1, NATr, ampR*	This study
pLC791	pLC49 *CAS5*^{S769E}*-HA-HIS1, NATr, ampR*	This study
pLC800	pLC49 *CAS5*^{S769A}*-HA-HIS1, NATr, ampR*	This study
pLC573	*pFA-TAP-ARG4, ampR*	[10]
pLC605	*CaTAr-FLP-CaNAT, ampR*	[11]
pLC818	pLC49 *CAS5-HA-HIS1, NATr, ampR*	This study
pLC857	pLC49 *CAS5*$^{S462E/S464E/S472E/S476E}$*-HA-HIS1, NATr, ampR*	This study
pLC858	pLC49 C*AS5S*$^{462A/S464A/S472A/S476A}$*-HA-HIS1, NATr, ampR*	This study

Supplementary Table 3. Oligonucleotides used in this study.

Name	Description	Sequence
oLC274	pJK863down-F	CTGTCAAGGAGGGTATTCTGG
oLC275	pJK863up-R	AAAGTCAAAGTTCCAAGGGG
oLC300	Tetp-F-NotI	ATAAGAATGCGGCCGCGTTTGGTTCAGCACCTTGTCG
oLC534	CaTAR-797-R	GATGGAGATAGTTTACGG
oLC600	JB-GFP+344-R	CCTTCAAACTTGACTTCAGC
oLC752	GPD1+570-F	AGTATGTGGAGCTTTACTGGGA
oLC753	GPD1+766-R	CAGAAACACCAGCAACATCTTC
oLC1593	TAP-R	TAAACTTTGGATGAAGGCG
oLC1594	ARG4-F	ATGTTGGCTACTGATTTAGCTG
oLC1645	HIS-F	ACAAACCTACTAATATCAGAT
oLC1752	CaEcm331 664-F	AACTTGACTAGTGTCAACGG
oLC1753	CaEcm331 961-R	CTTGGAAATCACCAGATACC
oLC2017	CaCas5-70F M13R	CTATTCTAATTTATTTACTTTGCTTTT CATCCCACCCCTTTGTTGGTAAATATAGAC TTTAACATATACTGGAAACAGCTATGACCATG
oLC2018	CaCas5+2536R M13F	AAAATACGAATTATCTATATGGATT ATACTTTAAATAATACCGTCTTTTAATG CATAGTCTATATAATGTGTAAAACGACGGCCAG
oLC2029	CaHA-R	GGCGAGGTATTGGATAGTTC
oLC2034	CaCas5-465F	GCTTGGATTTTCCCCCATTAG
oLC2035	CaCas5+3422R	GTTGTCATAATCCTACAGG
oLC2047	CaCas5+1349F	CCAATGACTTCATATCCACC
oLC2048	CaCas5+1559R	CCACCTGAAGTTGAATTGG
oLC2088	CaCAS5_pLC605F	CTATTCTAATTTATTTACTTTGCTT TTCATCCCACCCCTTTGTTGGTAAATATA GACTTTAACATATACTGGAAACAGCTATGACCATG
oLC2089	CaCAS5_pLC605R	GTAAACTATTTGTACCATCATCATA TGGCTGTGATAGCTGTGTCGGCGAAC TTAATAAATAATTCTCCATCGACTATTTATATTTGTATG
oLC2161	CaCAS5 HA-ARG4 F	AAAGATTCATGGACTTGTGAAAGG ACAAGAAGAGTTTACAAGAGTGTTGAAT GAAAACAAAGAAGTTTCCCCCGGGTACCCATACGATGT
oLC2162	CaCAS5 HA-ARG4 R	AAAATACGAATTATCTATATGGATTA TACTTTAAATAATACCGTCTTTTAATGCATAG TCTATATAATGTTCGATGAATTCGAGCTCGTT
oLC2163	CaCAS5+2276F	GACCAGAACATGTTAAACGTC
oLC2164	CaCAS5+2737R	GTCATCATGCGAGTTATGG
oLC2256	CaPGA13+468-F	CCAACCACTACATCTACTTC
oLC2257	CaPGA13+665-R	GGAACAACAACTGTAGTTGG
oLC2470	CaRLM1+868F	CCGGGAATGTTCCAAATACC
oLC2471	CaRLM1+1114R	GGAACTGTTGATACTGCTG

oLC3052	CaCAS5+1812-F	GGCTGTTGTTAAACAGGAAAAG
oLC3371	CaCAS5+1812-R	CTTTTCCTGTTTAACAACAGCC
oLC3424	CaSWI4+3144F TAP	TGGTGTTAAAGTTGAAGAAATTGAC AGTTTAATTGATGGAATTGCCGAATCATT AACTGAAGGTATGACGGGTCGACGGATCCCCGGGTT
oLC3425	CaSWI4+3286R HIS/ARG	CATCGAGTCAATTTAATAAAACTGTC CTCTTTCAATTTTGTTCCGATTTAATTTC CCCCATCTATCGTAATCGATGAATTCGAGCTCGTT
oLC3426	CaSWI4+2914F	GTTGAACAACATGAGTCAAG
oLC3427	CaSWI4+3581R	GTGTTTTCCCTCTGTTATTG
oLC3428	CaSWI6+2118F TAP	TACAAATGTTGGTGTAAACGAAGTT GATGAATTTTTAGACGGGTTGTTGGAAGC AGTGGAAGGACAACAGGGTCGACGGATCCCCGGGTT
oLC3429	CaSWI6+2260R HIS/ARG	GAATAAACATACAAAAGAATAGGA ATCGTTTTTTTTTTTGATTTTTTTTTGTTTGA GTTGGTGATATTGATCGATGAATTCGAGCTCGTT
oLC3430	CaSWI6+1739F	CCAATAATCGTTTCAACACC
oLC3431	CaSWI6+2844R	GTTGCAACAATGGTACAAAG
oLC3472	CaSWI4-70F M13R	GTTTGTATCACCCATATTTAATTTC ATTATATCCTTTTGATACTTCATTGATACTT AAAACTACTACATAGGAAACAGCTATGACCATG
oLC3473	CaSWI4+3286R M13F	CATCGAGTCAATTTAATAAAACT GTCCTCTTTCAATTTTGTTCCGATTTAA TTTCCCCCATCTATCGTAAGTAAAACGACGGCCAG
oLC3474	CaSWI4-988F	CGTTGATCACACTTCATTTC
oLC3490	CaGLC7-70F	TATATTTTTTTTTTTTTTTTATTTGGTG TGTCCAATTGCTTACTAACAACATTTAG TAATATAGCCTATAGGAAACAGCTATGACCATG
oLC3491	CaGLC7+1063R pLC49	AAACAGGAATAATAAAATAACAATAGT GACAATAATTTTATCAATTTCAAGACTC ATCGTGTAAACTCGAGTAAAACGACGGCCAG
oLC3492	CaGLC7+62R **pLC605**	TCCAAAAGACGGTCGACAATATTGTCAAC GGAAATGGAATCGTCTTCGATATTGTTTGACATCGACTA TTTATATTTGTATG
oLC3494	CaGLC7-1520R	GAAGAAAGTAGGTGTTGTTG
oLC3495	CaGLC7+237R	GAATAAACGGAGTAGATCG
oLC3558	CaGLC7-432F-KpnI	GGGGTACCGAAGAAGAATTGAGCGAGAG
oLC3561	CaGLC7+1431R-SacI	CCCGAGCTCGACTCTATATATGGTACAAC
oLC3641	CaSWI6A-70F M13R	CATACTTATTTCATTGAAAGGAAGCTGAACG TTATCATTATAAACGCTGGCTCAAGTTTATTAAC AAACTGGAAACAGCTATGACCATG
oLC3642	CaSWI6A+2191R M13F	GAATAAACATACAAAAGAATAGGAATCGTTTTTTTTTT TGTTTTTTTTTTGTTTGAGTTGGTGATATTGAGTAAAACG ACGGCCAG
oLC3645	CaSWI6-449F	CAGAAATGTGTACATGCTAG
oLC3654	CaSWI6+2660R	GCATTGAGTATAACCAGTAG
oLC3793	CaGLC7AB-320-F pLC605	AGTGAGTGAGAAAATTTTCTAATTAAACAAGAACAA AAAGGAAGGAAAAAAAAAAACACATCATTTTTTTGG

		AAACAGCTATGACCATG
oLC3794	CaGLC7AB-448-F	GGATGGAAAAAAAGAGGAAG
oLC4260	CaGLC7AB+392F	CAGTATCAACCGTATCTATG
oLC4261	CaGLC7AB+700R	CATCAGGTCCAAAAGTAAAC
oLC4417	yEmRFP+571R	AGCACCTGGTAATTGAACTGG
oLC4748	CaDAD2+314-F-linker-GFP	AAGAAGAAGCAGATGAAGAAGAAGGTGTTAGAGATAGTG AAGAAGTTGAAGAATCCACGGAAGGTGGTGGTTCTAAAGGTGAAGAATTAT
oLC4749	HIS-CaDAD2-R	GCCAGAATTTAGAGTTATTATATGTACACATTTTTTTAATATA AACATGTAGCAATATACTTTAGAATTCCGGAATATTTATGAGAAAC
oLC4751	CaDAD2+49-F	GCAAACTTGGAACGATTTAG
oLC4750	CaDAD2+525-R	CAACGAGTCATTGTTAAGTC
oLC4752	CaHHF1-pLC447-F	TTCATTGGATGTTGTTTACGCTTTGAAGAGACAAGGTAGAACCTTGTATGGTTTCGGTGGTGGTGGTGGTGTTTCAAAAGGTGAAGAAGA
oLC4753	CaHHF1-GFP/NAT-R	ACTTCTTAAATTAATACTATACAATAAAGAAAACGAACTAAAAAGACAATTAGAAATACAACCCAGTGTAAAACGACGGCCAGTGAATTC
oLC4754	CaHHF1+28-F	GGTAAAGGTTTAGGAAAAGG
oLC4755	CaHHF1+486-R	CATTTTACTAGGCAACTGAC
oLC5247	CaWSC1+443-F	CAATAACTGGATCTCCTAAC
oLC5248	CaWSC1+672-R	CTTTTCTGATTTCCCATCAG
oLC5259	CaWSC2+589-F	CCATCTACATCTACATCTTC
oLC5260	CaWSC2+839-R	GGATGTTGTTCCATTTCTTG
oLC5277	CaMCM2+964-F	CATCAAGAAGTTCACGTTAG
oLC5278	CaMCM2+1168-R	CAGTATTCGAATCTTGAACG
oLC5279	CaMCM3+1124-F	CCAAATCTCAAGTATTACGG
oLC5280	CaMCM3+1357-R	GTTCCATAACTTCGTGAATG

Supplementary References

1 Davis, D., Edwards, J. E., Jr., Mitchell, A. P. & Ibrahim, A. S. *Candida albicans RIM101* pH response pathway is required for host-pathogen interactions. *Infect Immun* **68**, 5953-5959 (2000).

2 Bruno, V. M. *et al.* Control of the *C. albicans* cell wall damage response by transcriptional regulator Cas5. *PLoS Pathog* **2**, e21, doi:10.1371/journal.ppat.0020021 (2006).

3 Noble, S. M. & Johnson, A. D. Strains and strategies for large-scale gene deletion studies of the diploid human fungal pathogen *Candida albicans*. *Eukaryotic cell* **4**, 298-309, doi:4/2/298 [pii]10.1128/EC.4.2.298-309.2005 (2005).

4 Shapiro, R. S., Zaas, A. K., Betancourt-Quiroz, M., Perfect, J. R. & Cowen, L. E. The Hsp90 co-chaperone Sgt1 governs *Candida albicans* morphogenesis and drug resistance. *PLoS One* **7**, e44734, doi:10.1371/journal.pone.0044734 (2012).

5 LaFayette, S. L. *et al.* PKC signaling regulates drug resistance of the fungal pathogen *Candida albicans* via circuitry comprised of Mkc1, calcineurin, and Hsp90. *PLoS Pathog* **6**, e1001069, doi:10.1371/journal.ppat.1001069 (2010).

6 Xie, J. L. *et al.* Signaling through Lrg1, Rho1 and Pkc1 Governs *Candida albicans* Morphogenesis in Response to Diverse Cues. *PLoS Genet* **12**, e1006405, doi:10.1371/journal.pgen.1006405 (2016).

7 Morschhauser, J., Michel, S. & Staib, P. Sequential gene disruption in *Candida albicans* by FLP-mediated site-specific recombination. *Mol Microbiol* **32**, 547-556 (1999).

8 Gerami-Nejad, M., Berman, J. & Gale, C. A. Cassettes for PCR-mediated construction of green, yellow, and cyan fluorescent protein fusions in *Candida albicans. Yeast* **18**, 859-864, doi:10.1002/yea.738 (2001).

9 Keppler-Ross, S., Noffz, C. & Dean, N. A new purple fluorescent color marker for genetic studies in *Saccharomyces cerevisiae* and *Candida albicans*. *Genetics* **179**, 705-710, doi:10.1534/genetics.108.087080 (2008).

10 Lavoie, H., Sellam, A., Askew, C., Nantel, A. & Whiteway, M. A toolbox for epitope-tagging and genome-wide location analysis in *Candida albicans*. *BMC Genomics* **9**, 578, doi:10.1186/1471-2164-9-578 (2008).

11 Leach, M. D. & Cowen, L. E. Membrane fluidity and temperature sensing are coupled via circuitry comprised of Ole1, Rsp5, and Hsf1 in *Candida albicans*. *Eukaryot Cell* **13**, 1077-1084, doi:10.1128/EC.00138-14 (2014).
